# Sodium propionate decreases implant-induced foreign body response in mice

**DOI:** 10.1371/journal.pone.0316764

**Published:** 2025-02-19

**Authors:** Deivenita Juliana Alves Carvalho do Carmo, Marcela Guimarães Takahashi Lazari, Letícia Cristine Cardoso dos Santos, Pedro Augusto Carvalho Costa, Itamar Couto Guedes Jesus, Silvia Guatimosim, Pedro Pires Goulart Guimaraes, Silvia Passos Andrade, Paula Peixoto Campos

**Affiliations:** 1 Department of General Pathology, Institute of Biological Sciences, Federal University of Minas Gerais, Campus UFMG, Belo Horizonte, Minas Gerais, Brazil; 2 Department of Physiology and Biophysics, Institute of Biological Sciences, Federal University of Minas Gerais, Campus UFMG, Belo Horizonte, Minas Gerais, Brazil; University of California, Merced, UNITED STATES OF AMERICA

## Abstract

The short-chain fatty acid (SCFA) propionate, beyond its actions on the intestine, has been able to lower inflammation and modulate angiogenesis and fibrogenesis in pathological conditions in experimental animal models. Its effects on foreign body reaction (FBR), an abnormal healing process induced by implantation of medical devices, have not been investigated. We have evaluated the effects of sodium propionate (SP) on inflammation, neovascularization and remodeling on a murine model of implant-induced FBR. Polyether-polyurethane sponge discs implanted subcutaneously in C57BL/6 mice provided the scaffold for the formation of the fibrovascular tissue. Fifteen-day old implants of the treated group (SP, 100 mg/kg for 14 days) presented a decrease in the inflammatory response as evaluated by cellular influx (flow cytometry; Neutrophils 54%; Lymphocytes 25%, Macrophages 40%). Myeloperoxidase activity, TNF-α levels and mast cell number were also lower in the treated group relative to the control group. Angiogenesis was evaluated by blood vessel number and VEGF levels, which were downregulated by the treatment. Moreover, the number of foreign body giant cells HE (FBGC) and the thickness of the collagenous capsule were reduced by 58% and 34%, respectively. Collagen deposition inside the implant, TGF-β1 levels, α-SMA and TGF-β1 expression were also reduced. These effects may indicate that SP holds potential as a therapeutic agent for attenuating adverse remodeling processes associated with implantable devices, expanding its applications in biomedical contexts.

## Introduction

Synthetic matrices have been used widely to replace and/or repair biological tissues. However, despite the benefits and advances of this medical procedure, adverse healing (excessive fibrosis, foreign body reaction- FBR) in the implant-host interface very often occurs, impairing implant device durability and functionality [1–7]. This reaction that begins after the interaction between the damaged tissue and the biomaterial is characterized by a sequence of overlapping events similar to wound healing (inflammation, granulation tissue formation, angiogenesis, remodeling). Neutrophils and mast cells, the predominant cell populations at the site of implantation for the first 2 days, degranulate and secrete inflammatory mediators (acute phase). In sequence, macrophages that differentiate from infiltrating monocytes become predominant. The activated macrophages stay at the implantation site releasing cytokines that stimulate migration and proliferation of endothelial cells and fibroblasts, responsible for angiogenesis and matrix remodeling, respectively. Fusion of macrophages takes place at the site of implantation leading to the formation of foreign body giant cells (FBGC), key components of FBR. Fibroblasts that appear around day 7 after implantation increase in number progressively, being responsible for the formation of a fibrotic collagenous capsule around the biomaterial. The combination of FBGC and collagenous capsule provides a barrier isolating the implants from the surrounding tissues which is detrimental to the implants’ function, safety, and biocompatibility [2, 8–11].

Several approaches have been considered to reduce FBR especially, capsule formation. These include optimization of physical properties and formulation of the biomaterial, as well as drug-based interventions. The use of steroidal and nonsteroidal anti-inflammatory and anti-fibrotic drugs, currently used to overcome the adverse FBR, is clinically limited. This indicates the need for novel therapeutic approaches [12–16].

Short-chain fatty acids (SCFAs), major energy substrates for colonocytes and maintenance of intestinal homeostasis and functions, have been shown to modulate different cell processes in other tissues outside the gut such as the immune system and the vasculature. They have also been shown to regulate either positively or negatively key components during healing/scar processes (inflammation, angiogenesis and matrix remodeling) [17–21]. Our research group has previously reported the effects of sodium butyrate on key components of the fibrovascular tissue induced by polyether-polyurethane sponge discs in mice [21–23]. There is also evidence that propionate and/or the synthetic derivative sodium propionate (SP) has been able to modulate inflammation, tissue damage, angiogenesis and fibrosis in several disease models. For example, SP reduced inflammation in the carrageenan-induced rat paw inflammation and decreased spinal cord trauma [24]. Propionate was shown to improve dextran sodium sulfate-induced colitis in a mouse model by reducing inflammation and oxidative stress [25]. Wang et al. [26] reported the protective effects of propionate against lipopolysaccharide-induced mastitis by suppressing the inflammatory response. The effects of propionate on angiogenesis were reported in a model of bronchopulmonary dysplasia in neonatal mice [27]. Propionate was also shown to reduce cardiac fibrosis/remodeling in hypertensive mice [28].

Given these range of modulatory actions of SP on multiple targets in various physiopathological conditions and considering the prominent role of inflammation, angiogenesis and fibrogenesis in the FBR, we hypothesized that the SCFA derivative, SP might modulate these components in our murine model of FBR induced by synthetic matrix of polyether-polyurethane. To test our hypothesis, we treated C57BL/6 mice bearing subcutaneous implants with SP and analyzed the inflammatory response, angiogenesis, foreign body giant cell formation and fibrotic capsule. Our findings reveal the relevant role of SP in controlling adverse healing.

## Materials and methods

### Ethics statement

The use of animals and procedures for this study was approved by the Ethics Committee of Animal Experimentation (CEUA) of Federal University of Minas Gerais, (protocol number 282/2018) and all applicable international, national, and/or institutional guidelines for the care and use of animals were followed. All surgery was performed under anesthesia, and all efforts were made to minimize suffering.

### Animals

C57BL/6 mice 8 weeks of age (20-25g body weight n = 70) provided by the CEBIO (Animal Unit Center–UFMG/Brazil) were used. All animals had free access to water and standard feed (NUVILAB CR-1 Brazil). They were housed with light cycles of 12 hours and temperature controlled. All procedures maintained the standards established in the guidelines for the care and use of experimental animals by the Institutional Committee for Animal Welfare and in accordance with the Ethics Committee on Animal Use Research of UFMG.

### Sponge discs implantation and sodium propionate (SP) treatment

Polyether-polyurethane sponge discs (Vitafoam Ltd. Manchester, UK) 8 mm in diameter and 5 mm thick, were used as the implanted material to provide the scaffold for fibrovascular tissue growth. The sponge discs were immersed in ethanol 70% v/v overnight, with subsequent sterilization by boiling in distilled water for 30 minutes. Before to sponge implant surgery, the mice were anesthetized by intraperitoneal injection of the ketamine (150 mg/kg) and xylazine (10 mg/kg) mixture, trichotomized and skin antisepsis with ethanol 70% v/v was performed [22]. The sponge disc was implanted subcutaneously on the dorsum of the mice and the incision was closed with no absorbable suture. After surgery, the mice were housed in individual boxes, with a normal diet and free water. Post-operatively, the animals were monitored for any signs of infection, discomfort or distress.

The mice were divided into two groups of 35 animals, the control group received 100 μL of filtered water by gavage, daily for 14 days. The treated group received 100 mg/kg sodium propionate (Sigma; 96.06 g / mol) in 100 μL of filtered water [24]. At the 15th day post implantation, the animals were humanely killed by an overdose of the anesthetic. The implants were carefully removed, photographed, weighed and processed for analysis. The images of the implants were captured with a stereo microscope (Nikon SMZ725T), equipped with Qcapture Pro 7 software, with a magnification of 6.5x and 50x to evaluate the aspect of the post-removal implant and the formation of vessels on the implant surface.

### Histological staining and morphometric analysis

The implants of five animals per group were fixed in 10% formaldehyde for 24 h. After inclusion of paraffin, the sections with thickness of 5 μm were processed and stained with hematoxylin and eosin (H&E), Dominici’s method and Picrosirius Red to evaluate the intra-implant tissue.

The images of slides stained with hematoxylin and eosin were used to quantify the number of blood vessels, giant cells and measurement of capsule thickness. All fields with these structures were analyzed. For the quantification of mast cells, we used slides stained with Dominici’s method. The images were captured with a digital camera (CoolSnarp-Pro) connected to an Olympus BX51 microscope equipped with Image-Pro Express 7.0 software (Media Cybernetics, USA) with a resolution of 2560 x 1920 pixels, with a magnification of 400x and 1000x. Images of picrosirius-stained slides on average 50 fields per slide were captured with a camera with polarized light (Spot Insight) connected to an Olympus BX41 microscope equipped with Spot 3.4 software, with a resolution of 2560 x 1920 pixels, with a magnification of 200x. To quantify Picrosirius Red staining, we used Image Pro Plus 7.0 software.

### Dosage of myeloperoxidase levels

The assessment of neutrophils into implants (n = 8–10) was made indirectly by quantifying myeloperoxidase (MPO) activity, a lysosomal hemoprotein found in the azurophilic granules in neutrophils, as previously described [29]. Pellets from centrifugation of implants homogenates were divided into two portions, a part of the corresponding pellet was weighed, homogenized in pH 4.7 buffer (0.1 M NaCl, 0.02 M NaPO4, 0.015 M NaEDTA), and centrifuged at 12,000 x g for 10 min. The pellets were then re-suspended in 0.05M NaPO4 buffer (pH 5.4) containing 0.5% hexadecyltrimethylammonium bromide (HTAB) followed by three freeze thaw cycles using liquid nitrogen. MPO activity in the supernatant samples was assayed by measuring the change in absorbance (optical density; OD) at 450 nm using tetramethylbenzidine (1.6 mM) and H2O2 (0.3 mM). The reaction was terminated by adding 50 ml of H2SO4 (4 M). Results are expressed as a change in OD per gram of wet tissue.

### Measurement of intra-implant content of VEGF, TNF-α and TGF-β1

Production of cytokines was determined by Immunoassay Kits (R&D Systems, USA) according to the manufacturer’s protocol, in the supernatant from each implant (50 μl). The implants were homogenized in PBS pH 7.4 containing 0.05% Tween, and centrifuged at 10,000 x g for 30 min. Briefly, dilutions of cell-free supernatants were added in duplicate to ELISA plates coated with a specific murine monoclonal antibody against the cytokine, followed by the addition of a second horseradish peroxidase-conjugated polyclonal antibody, also against the cytokine. After washing to remove any unbound antibody-enzyme reagent, a substrate solution (50 μL of a 1:1 solution of hydrogen peroxide and tetramethylbenzidine 10 mg/ml in DMSO) was added to the wells. Color development was halted after 20 min incubation with 2 N sulfuric acid (50 μL) and the intensity of the color was measured at 492 nm on a spectrophotometer (Varisoskan Flash 40053). Standards were 0.5-log10 dilutions of recombinant murine cytokines from 7.5 ρg ml-1 to 1000 pg ml-1 (100 μl). The threshold of sensitivity for each chemokine is 15.625 pg/mL. The results are expressed as pg cytokine per mg wet tissue (implants).

### Flow cytometry analyses

Intra-implant leukocytes were quantified 15 days post- implantation by flow cytometry. The following monoclonal panel of fluorescent antibodies were used: anti-CD45 (fluorochrome Pe-Cy5), anti-CD11c (fluorochrome/eF450), anti-CD3 (fluorochrome PE), anti-F4/80 (fluorochrome FITC), anti-MHC-II (fluorochrome Cy7PE), anti-GR-1 (fluorochrome APC). Ten sponge implants from each group were used. The implants were shred with scissors in 1 ml of HBSS, then 2.5 ml of filtered and sterilized type 4 collagenase (Sigma Chemicals, St Louis, MO, USA) and trypsin were added to the fragments. After incubation for 30 min at 37° C, the cells were washed and centrifuged (500 g for 10 min at 4° C). The cells isolated from sponges were suspended in a staining buffer (0.1% BSA in PBS). Subsequently, the cells were washed with FACS buffer, flushed to surface molecules for 20 minutes at 4° C. At least 50,000 blocked events were acquired for analysis using FACSCanto-II (BD Biosciences, San Jose, CA, USA).

The data were analyzed using FlowJo Version 9.7.5 (TreeStar, Carrum Downs, Australia). Direct dispersion (FSC-A) and lateral dispersion (SSC-A) were used to initially remove debris and capture leukocytes. Leukocytes were quantified based on CD45 expression, and then T lymphocytes were quantified based on CD45 + CD3 expression. From the CD45+ cells, the expression of GR1 versus F4 / 80 was evaluated to select monocytes (GR1 + and GR1Low) and neutrophils (GR1 + F4 / 80Neg). A more detailed analysis of monocyte subpopulations was based on the expression of GR1 and here designated as: inflammatory monocytes (F4 / 80 + CD11b + GR1-High) and patrolling monocytes (F4 / 80 + CD11b + GR1-Low). The F4/ 80 High GR1Low/Neg port was used to characterize macrophages.

### Western blot

Western blot was performed using another set of sponge disc (n = 10 for each group) which were dissected and immediately frozen in liquid nitrogen. The frozen implants were homogenized in ice-cold lysis buffer (in mmol/L: 100 NaCl, 50 Tris-base, 5 EDTA 2H2O, 50 Na4P2O7 10 H2O, and 1 MgCl2, pH 8.0) containing 0.3% Triton X-100, 1% Nonidet P40, 0.5% sodium deoxycholate, and 20 mM NaF, enriched with protease and phosphatase inhibitors cocktail. 50 μg of protein were separated by SDS-PAGE followed by western blotting, as described previously [30]. Primary antibodies and their sources are as follows: anti-α-SMA (1:1000, Abcam, #ab21027), TGF-β1 (1:1000, Abcam, #ab9758) and GAPDH (1:3000, Santa Cruz Biotechnology, #sc-32233). Immunodetection was carried out using enhanced chemiluminescence detected with LAS 4000 equipment (GE HealthCare Life Science). Protein levels were expressed as a ratio of optical densities. GAPDH was used as a control for any variations in protein loading.

### Statistical analysis

Results are presented as mean ± SEM or median. Normality and homoscedasticity were determined for further statistical analysis. Statistical analysis was performed using GraphPad Prism 7.0 software, and a value of p⥶0.05 was considered statistically significant. Comparisons between three or more groups were made using oneway analysis of variance (ANOVA) followed by the Newman-Keuls correction factor for multiple comparisons as a post-test. Comparisons between the two groups were made by Student’s t-test or Mann Whitney unpaired groups.

## Results

**Fig 1:**
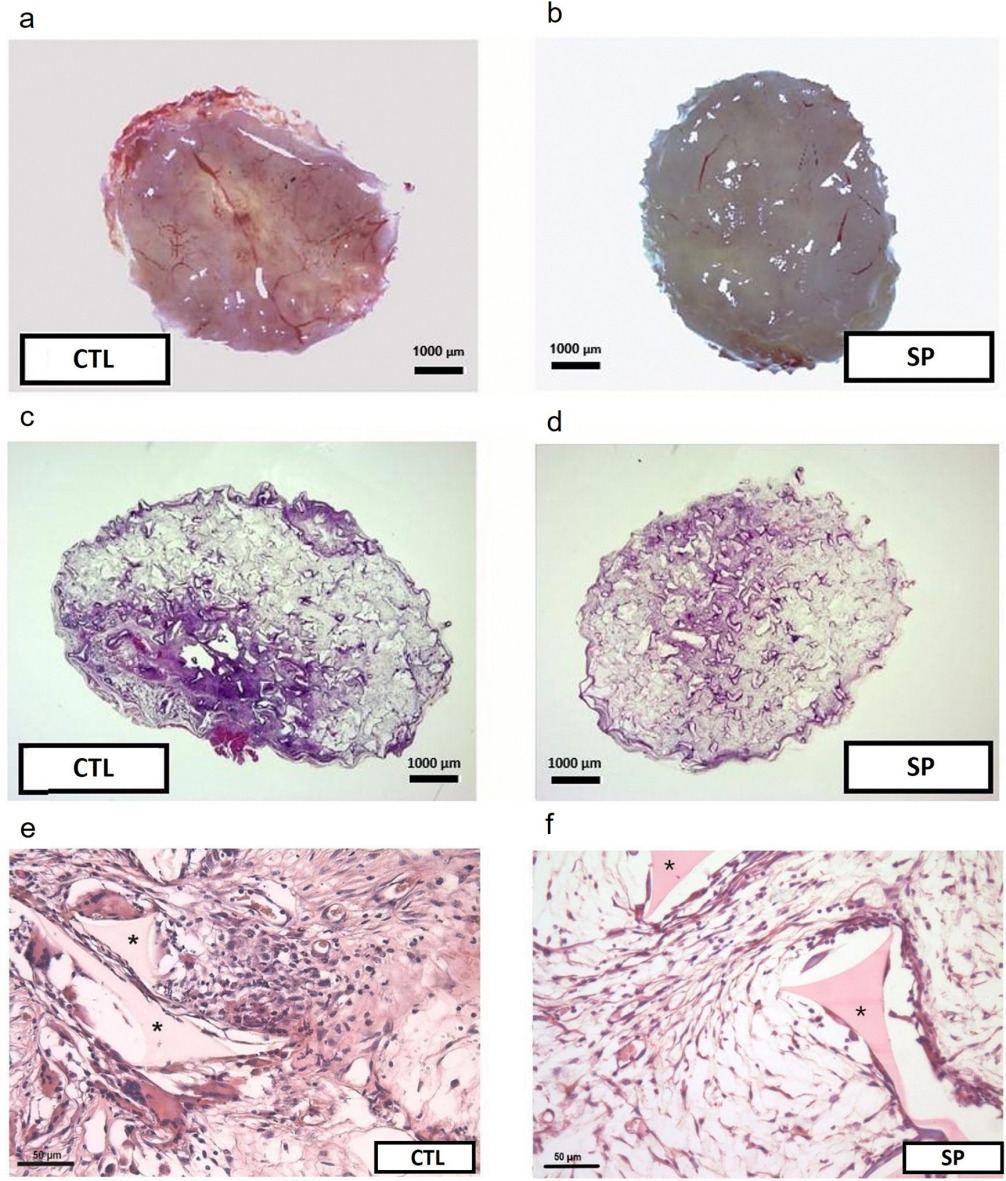
Macroscopic view of the implant after removal versus histological image in H&E. Photomicrography of the sponge implant 15 days post implantation in (a) and (b), from the control and treated groups, respectively. In (c) and (d), histological images in H&E (6.5x magnification) of the control and treated groups, respectively. In (e) and (f) representative photomicrographs of the implants (H&E), 400x magnification. Differences in tonalities and in the amount of tissue occupying the porous of the implants between the groups are observed. The polyurethane polyether sponge has a triangular appearance marked in asterisk in the image. Scale bar 1000 μm and Scale bar, 50 μm.

Oral administration of SP (100 mg/kg, for fourteen consecutive days) showed no signs of toxicity in the mice, such as sedation, weight loss, or changes in motor activity. Macro and histological analyses (H&E) of the implants of control and treated animals showed fibrovascular tissue formation inside the sponge matrix 15 days post-implantation. The porous synthetic implants became full with inflammatory components (exudate and cells) and granulation tissue containing newly formed blood vessels, multinucleated FBGCs, extracellular matrix and fibroblasts-like cells. These features were lower in implants of treated animals (Fig 1A–1F).

**Fig 2:**
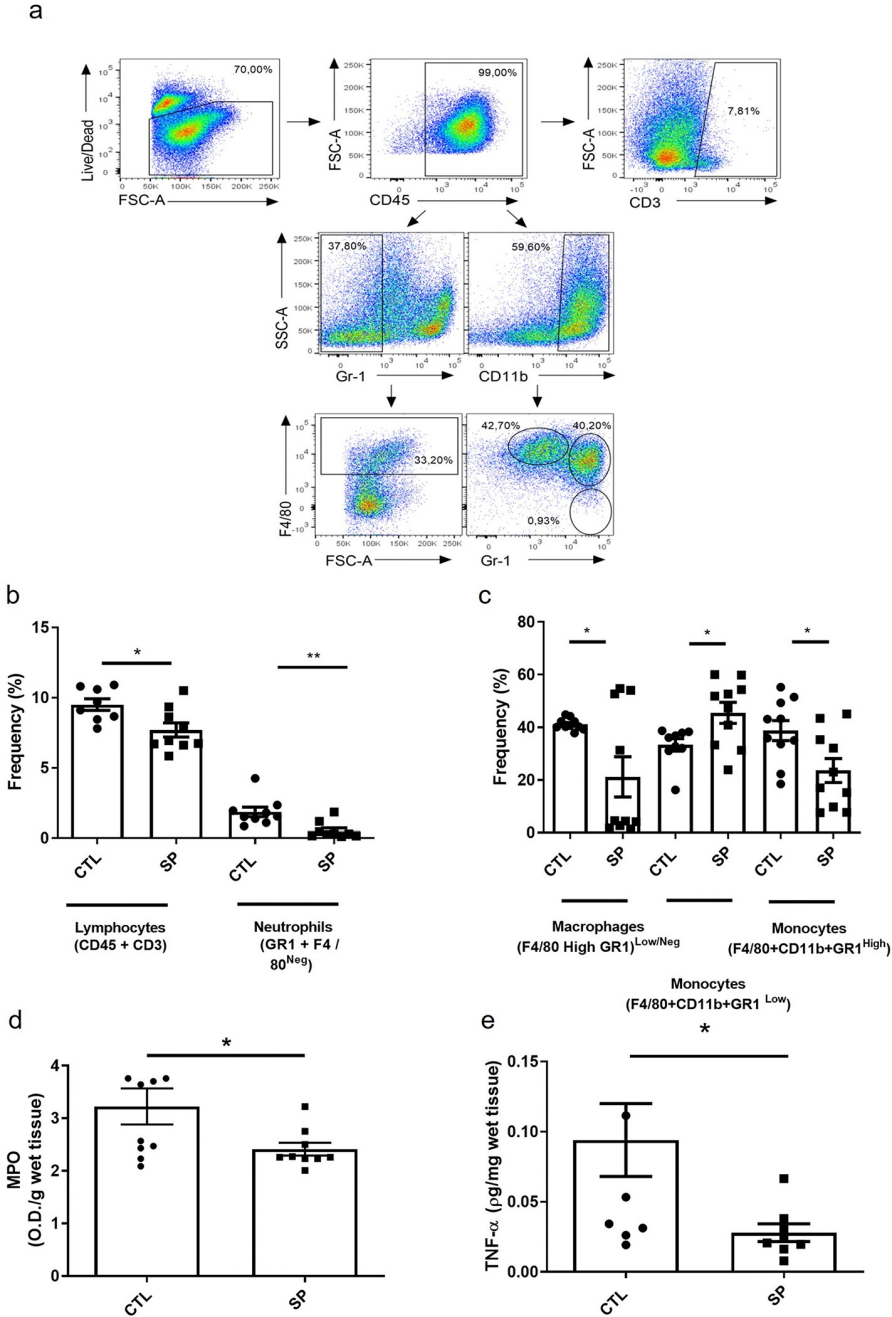
Effects of sodium propionate on implant cellularity, myeloperoxidase activity (MPO) and TNF-α levels 15 days post implantation. Represented in (a) is the strategy of analysis in dot plots identifying the infiltration of T lymphocytes, neutrophils, inflammatory monocytes, patrolling monocytes and macrophages inside the implant. In (b), the frequency of these cells was compared in both groups. In (c) and (d), the results of the quantification of myeloperoxidase and TNF-α levels in both groups. The results are expressed as means ± SEM. *p < 0.05 **p < 0.01; ANOVA, Student’s T test, n = 8–10 animals per group.

We assessed the inflammatory response of the implants by quantifying cellular influx, inflammatory enzyme activities, TNF-α levels and mast cell number. All inflammatory markers examined were decreased in implants of SP-treated animals. The treatment was able to decrease inflammatory cells influx; neutrophils 54%; lymphocytes 25%, macrophages 40%, relative to control implants. MPO activity and TNF-α levels were also reduced by 24% and 50%, respectively in the treated group (Fig 2A–2E).

**Fig 3:**
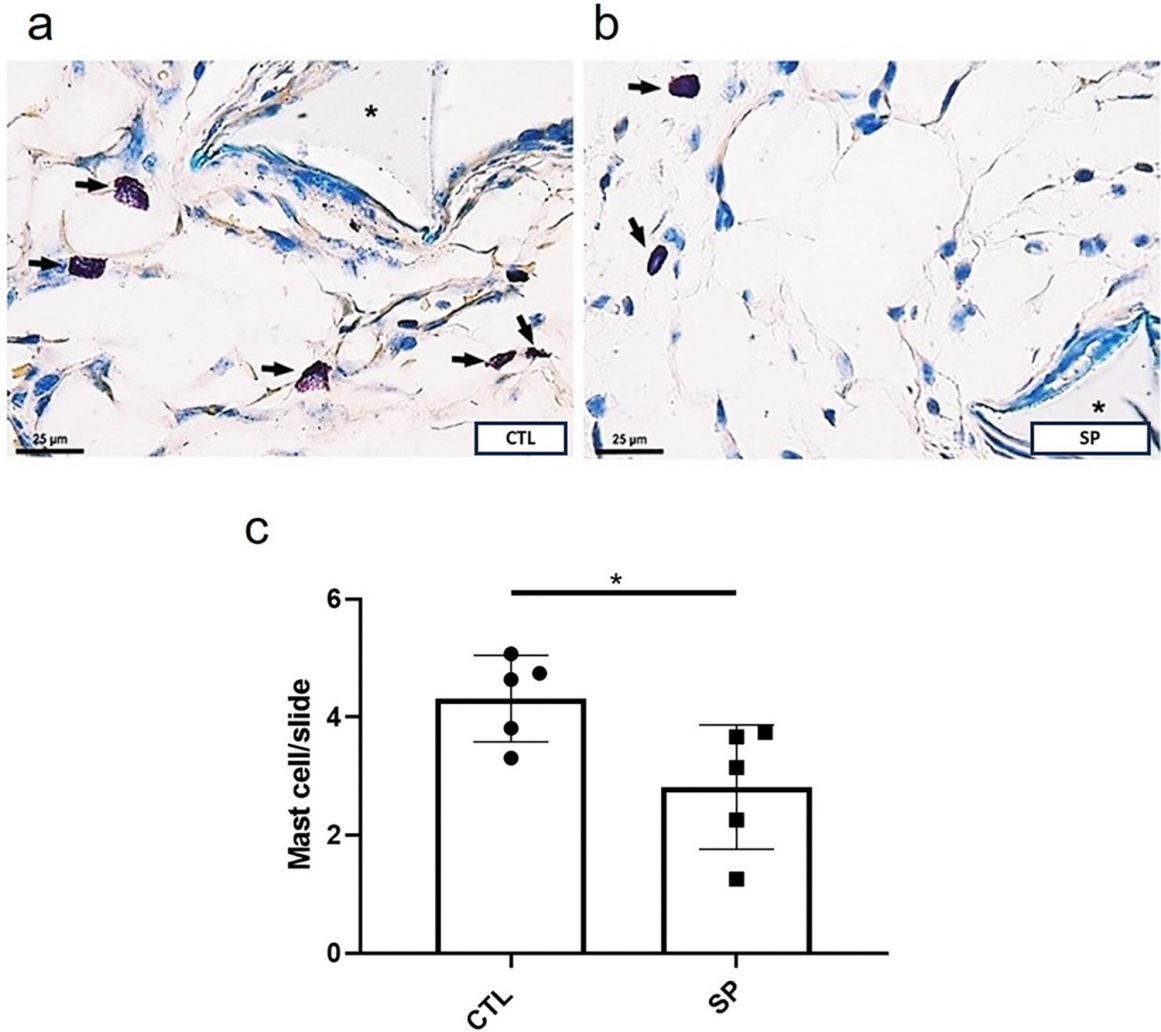
Effect of sodium propionate on mast cells infiltration. Photomicrograph of histological sections stained with Dominici. Mast cells are pointed out by the arrows, the control group represented in (a), and the treated group represented in (b). The results are expressed in (c), mean ± SEM. *p < 0.05; Student’s T test, n = 5 animals per group.

Another important cell type in the formation of the fibrovascular tissue are mast cells. The number of this cell population, as detected in implants stained with Dominici, decreased in implants of SP-treated mice compared with the control group (mast cell number in control group 4.0±0.3 versus SP-treated group 3.0±0.3, (Fig 3A–3C).

**Fig 4:**
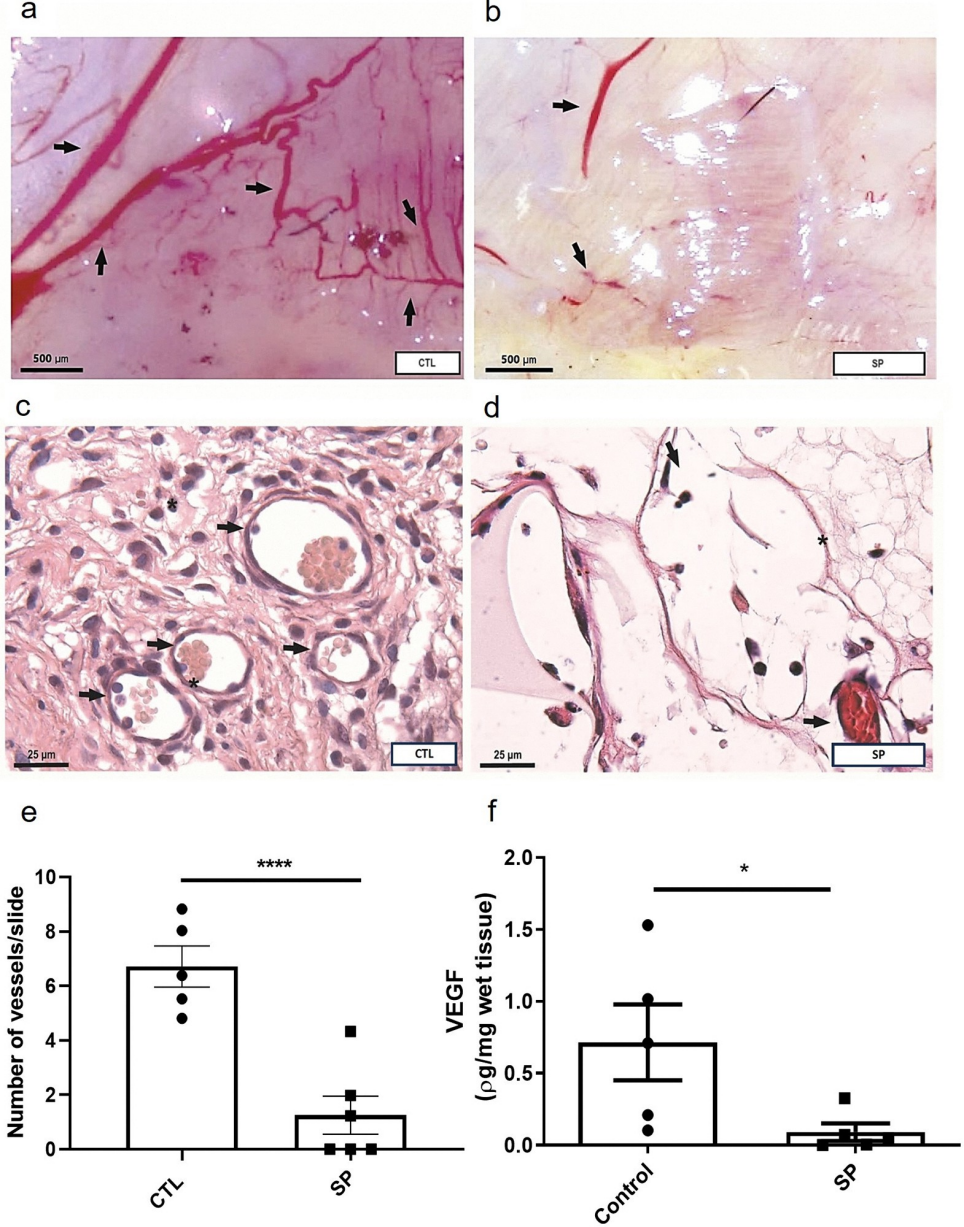
Effects of sodium propionate on angiogenesis. In (a) and (b) representative photomacrography (50x), showing newly formed blood vessels dispersed in the implant surface of the control animal in (a). Rare vessels on the surface of the implant of the animal treated with SP are seen in (b). Photomicrographs of the sponge implant stained with H&E are shown (c) and (d); magnification 1000x). In (e) and (f), the number of blood vessels and VEGF levels are shown. Arrows: intra-implant vessels. The results are expressed as means ± SEM. ****P < 0.0001; *P < 0.05, Student T test, n = 5 animals per group.

The other parameter analyzed and quantified was angiogenesis. Reduced vascularization in the macro images (Fig 1A and 1B) of the implants of SP-treated animals, was confirmed by the decrease in number of vessels (histological analysis H.E.). In the treated group the number was 1.26 ± 0.7 versus 6.7 ± 0.7 control group. The levels of VEGF, a potent pro-angiogenic mediator, were also decreased in the SP-treated group 0.09 ± 0.06 versus control group 0.71± 0.27 (Fig 4A–4F).

**Fig 5:**
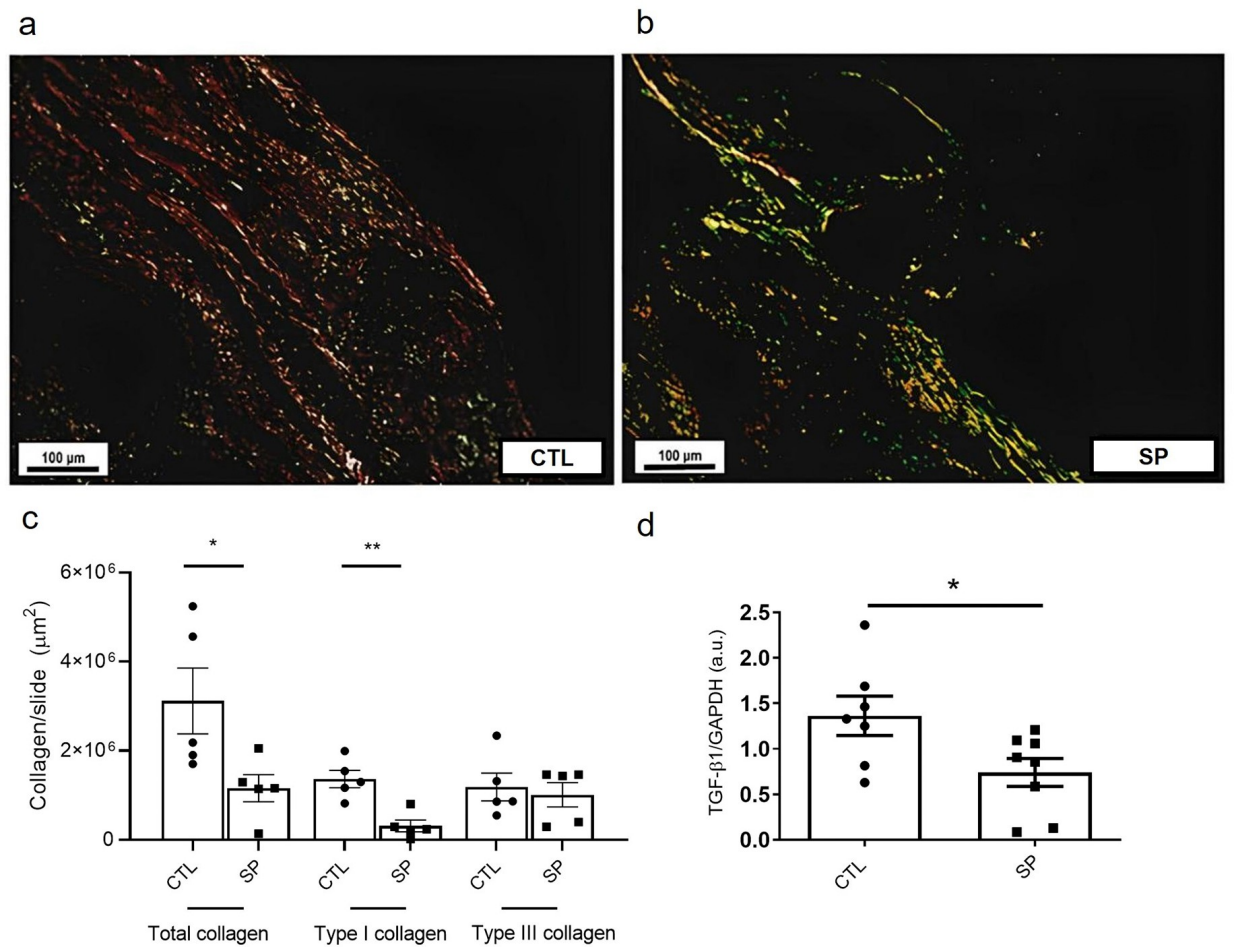
Effect of sodium propionate on collagen fiber deposition and TGF-β1 levels. Representative photomicrography of intra-implant collagen fibers evidenced by Picrosirius red staining, control group in (a), and SP group in (b). In this polarized light staining, type I collagen fibers acquire increased birefringence in bright yellow, orange or red hues, and collagen fibers type III acquire increased birefringence in green hues. Total collagen fiber quantity and Col I: Col III ratio are shown in (c; n = 5 animals per group). TGF-β1 levels intra implant are shown in (d) (n = 8–10). The results are expressed as means ± SEM *p < 0.05, **p < 0.01; Student T test.; mean± SEM *p < 0.05. Student T test).

Additionally, SP treatment was able to inhibit the FBR markers compared with the control. At day 15 post implantation, the amount of collagen (picrosirius staining) in the SP-treated group was less compared with the control group. In the control group the mean was 3118482 ± 740566 versus 1156159 ± 305114 μm^2^ in the SP group. In this group the predominant collagen fiber was type III 1009974 ± 271215 μm^2^, whereas in the control group it was 1183805 ± 313505 μm^2^. TGF-β1 levels intra-implant of treated animals were also lower 0.4 ± 0.08 than in the control group 1.3 ± 0.4 (Fig 5A–5D).

**Fig 6:**
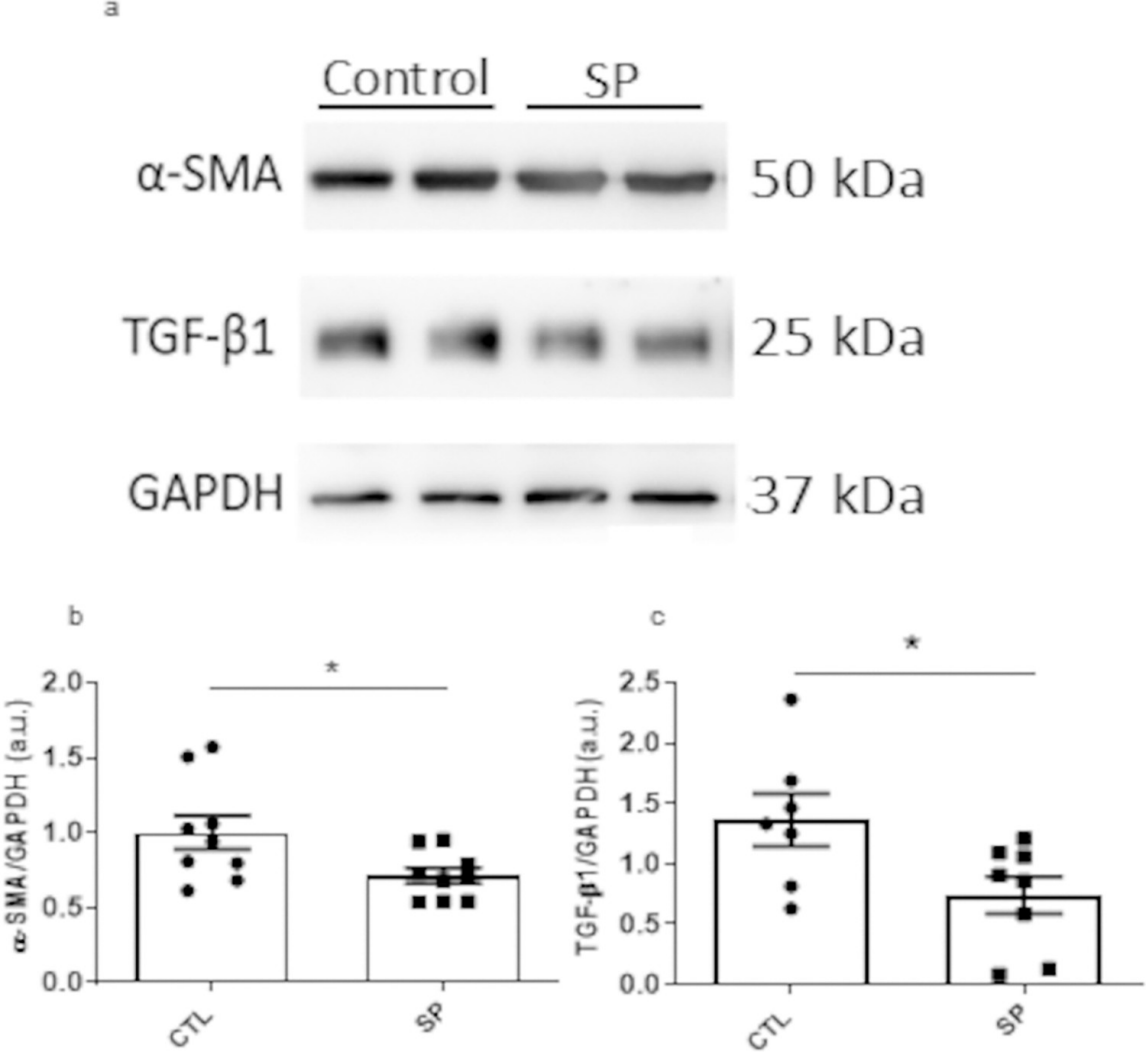
Effects of oral administration of SP on activation of α-SMA and TGF- β1 in implants. SP decreased the activation of α-SMA pathway (a-c) In (a), representative images of protein bands in each group. The results are expressed as mean ± SEM *p < 0.05, Student t test; n = 8 animals per group.

In addition, the effects of SP on the fibrogenesis axis was assessed by quantifying the activation of α-SMA and TGF-β1 by western blot. Alpha-smooth muscle actin (α-SMA, actin isoform), present in myofibroblasts was evaluated. Decrease in the relation α-SMA/TGF-β1 was observed in the SP group. α-SMA values in this group were 0.7 ± 0.05 versus 1.0 ±0.1 in the control group. TGF-β1 values in the treated group were 0.74±0.15 versus 1.3 ± 0.2 in the control group (Fig 6A–6C). These results indicate reduced fibrogenesis in implants of SP mice compared with the control group.

**Fig 7:**
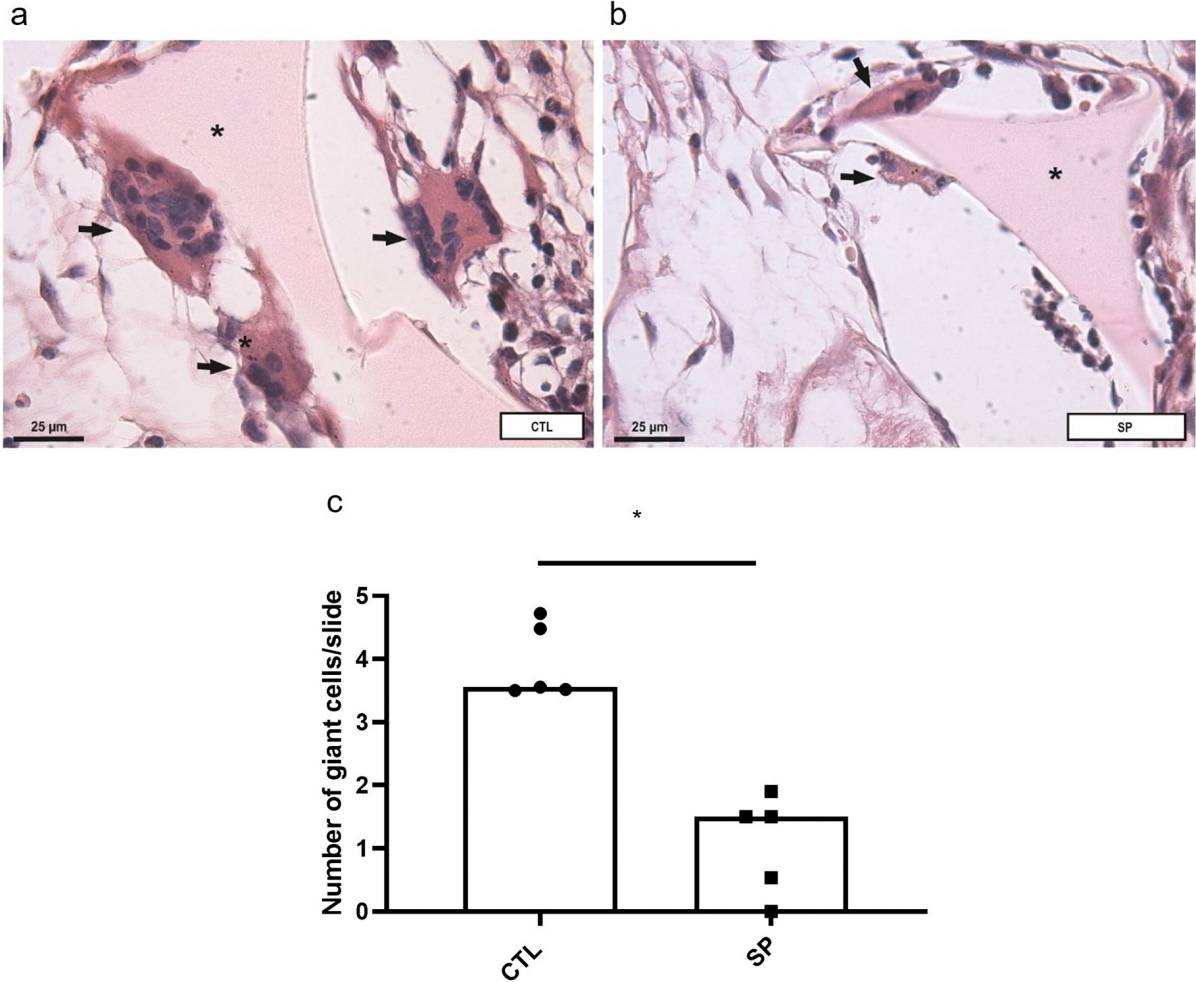
Effect of sodium propionate on foreign body reaction. Representative photomicrograph of the foreign body-like reaction stained with H&E in (a) in the control group, and in (b) in the SP group. The presence of multinucleated giant cells adhered to the biomaterial (pointed at asterisk) is observed in both groups. There is a very significant reduction in intra-implant cellularity and quantitative giant/field cells, represented in (c). It was observed morphological alteration of giant cells in the treated group, with reduction in the number of nuclei and cytoplasm. The results are expressed with the median; Mann Whitney test, n = 5 animals per group.

**Fig 8:**
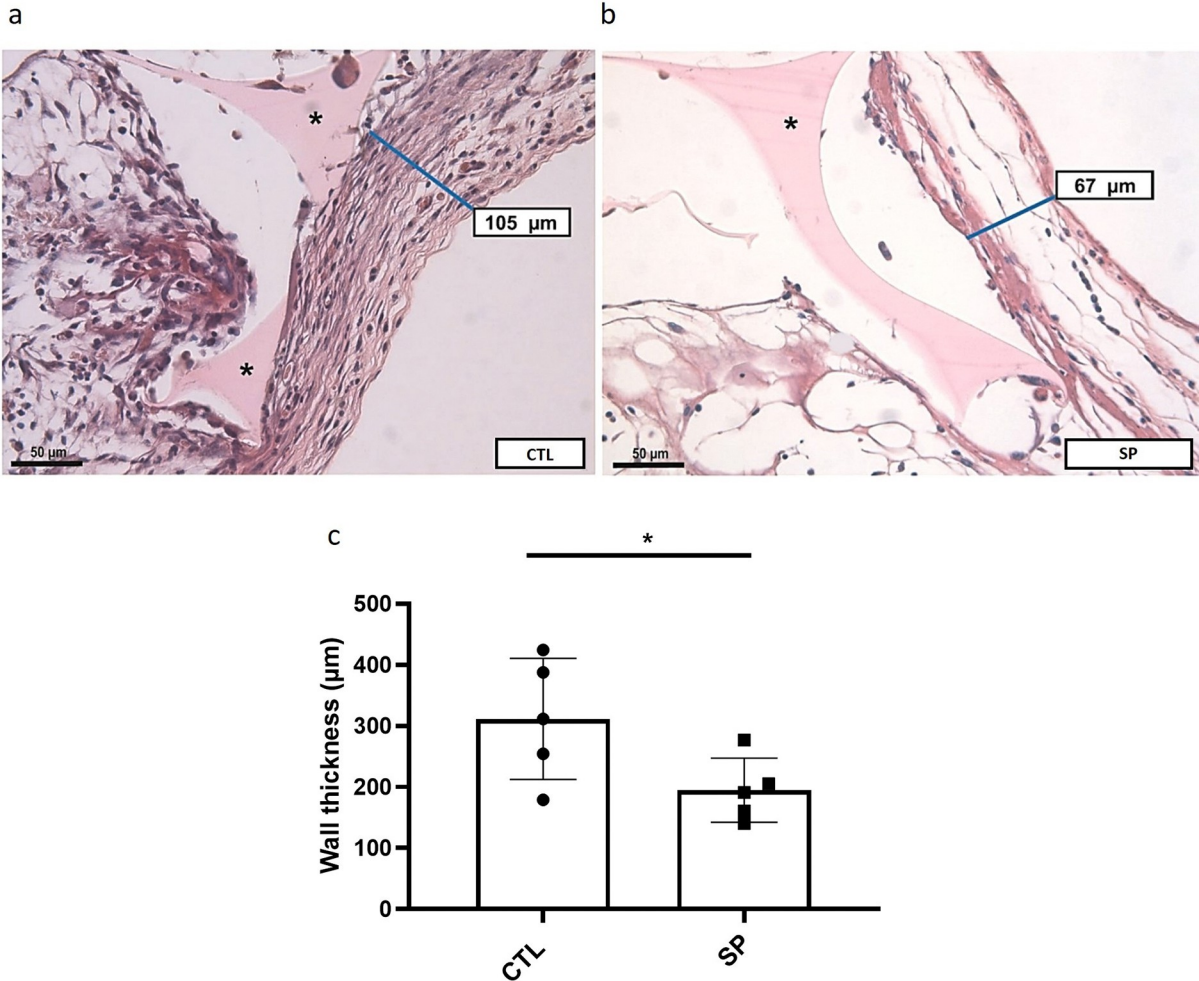
Effect of sodium propionate on capsule formation. Representative photomicrograph of the fibrous capsule involving the implants (H.E staining; (a), control group; (b), treated group). Measurement of capsule thickness was expressed in (c). The results are expressed as mean± SEM., *p < 0.05, Student’s T test, n = 5 animals per group.

Likewise, FBGC number decreased in the treated group median = 1.5, compared with the control group median 3.6 (Fig 7A–7C). There was also a decrease in wall thickness in the SP-treated group 194.8 ± 23.5 compared with the control 311.5 ± 44.4 (Fig 8A–8C).

## Discussion

The success in implanting a biomaterial is related to the intensity of the inflammatory reaction generated and its products. After insertion, the implants trigger a cascade of pro-inflammatory signaling that can evoke an intense foreign body-like inflammatory response with fibrovascular tissue formation that adheres to the implant interface and infiltrates into its compartments. This, in turn, very often leads to impairment of implant functionality, durability and proposed treatment [5, 22, 29, 31, 32]. A number of therapeutic strategies has been proposed to control the intensity of the inflammatory reaction to foreign body devices. Drug-based interventions, particularly, use of steroidal and nonsteroidal anti-inflammatory and anti-fibrotic drugs have been proposed to overcome the adverse FBR [12–15]. However, current treatments have failed to completely prevent/attenuate excessive FBR, indicating the need of novel therapeutic approaches [1, 22].

Studies involving natural compounds have shown that SCFAs, particularly, butyrate and propionate can act in the migration of leukocytes to inflammatory sites, inhibiting the expression of adhering molecules in a number of pathological processes [33–35]. Our research group has previously reported that sodium butyrate attenuated key components (inflammation, angiogenesis and fibrogenesis) of the fibrovascular tissue induced by the synthetic matrix of polyether-polyurethane implants in mice [22, 23, 36]. Given that we found no study that evaluated the effects of sodium propionate (SCFAs derivative) on the foreign body response, our aim in this study was to investigate whether this compound would attenuate the adverse remodeling induced by sponge implants in mice. Using the polyether-polyurethane implant model (sponge implant model in mice) we confirmed that the newly formed fibrovascular tissue that formed in and around the implants, during the time period examined (15 days), exhibited characteristics compatible with a foreign body type inflammation. We have identified the presence of neutrophils, mast cells, monocytes, lymphocytes, fibroblasts, macrophages and giant foreign body cells and intense neovascularization composing the fibrovascular tissue induced by the synthetic matrix. Furthermore, fibrous capsule, a hallmark of foreign body reaction, was observed enveloping the implants. Our results showed that sodium propionate administered orally, overall downregulated the parameters analyzed involved in the formation of the foreign body type response. It is particularly relevant to point out that the success of any treatment to attenuate the adverse response depends on the intensity of the FBR that in turn, is determined by various factors including; the chemical nature of the material, the type of implants (solid or porous scaffolds, insulin pumps, continuous glucose monitoring sensors, polymer-coated stents, breast implants, pacemaker, and others [37]. Thus, whether the effects observed in our study may be seen in other experimental models using, for example, solid implants, remains to be investigated.

In the first set of results, the inflammatory components of the implants were analyzed. Using flow cytometry, our results showed that SP was able to decrease the frequency/influx of leukocyte populations (neutrophils, lymphocytes and macrophages) by approximately 50%, relative to control implants. MPO activity and TNF-α levels were also reduced in the treated group. In addition, SP treatment reduced the number of mast cells, a key player in the foreign body response [38]. Our results are in agreement with some publications that showed reduced inflammation in the carrageenan-induced rat paw inflammation and decreased spinal cord trauma after treatment with SP [24]. Propionate was shown to improve dextran sodium sulfate-induced colitis in a mouse model by reducing inflammation and oxidative stress [25]. Wang et al. [26] reported the protective effects of propionate against lipopolysaccharide-induced mastitis by suppressing the inflammatory response. In these different models, SP acted inhibiting neutrophil activation.

Neutrophils are important cells in the coordination and intensification of inflammatory reactions in the acute and chronic phase of the host response to the biomaterial. They act by releasing granular proteins, reactive oxygen and chemokine species, and forming extracellular neutrophils traps (NETs) [39–41]. Studies observed that by inhibiting the migration of neutrophils in the acute phase of inflammation, and consequently reducing the concentration of their products in the inflammatory site, the recruitment of other leukocytes is compromised [42–44]. Neutrophils are also reported to be involved in the immune response to biomaterials. They release NETS when unable to phagocyte a harmful stimulus, and this action is considered an analogue of the formation of giant foreign body cells. The release of NETs increases the fibrogenic response, contributing to an excessive production of a dense fibrotic matrix, promoting fibrotic encapsulation and impairing tissue regeneration [40, 45–47] Thus, it is possible that, at least in part, reduced recruitment/activation of neutrophils by SP has contributed to downregulation of adverse remodeling response induced by the implants.

Lymphocytes are important leukocytes in foreign body response due to their role in the production of pro-inflammatory cytokines that sustain and orchestrate inflammatory events and promote the adhering and fusion of macrophages in giant cells [45, 48, 49]. In our study, a decrease in lymphocytes influx in the treated group was observed compared with the control group. It has been reported that SCFAs can alter phenotypic differentiation and lymphocyte function. Propionate has been shown to inhibit the activation and formation of T lymphocyte colonies at high *in vitro* concentrations [42, 50, 51].

Monocytes/Macrophages exert a relevant role in the FBR. The continuous stimulus caused by the permanence of the tissue/implant interaction evokes a chronic inflammation, with predominance of monocytes and macrophages and their different phenotypes in the inflammatory site [2, 22]. When stimulated and/or present in a hostile environment they form "alternatively activated macrophages" (M2) or foreign body multinucleated giant cells [52]. In our study, SP had distinct effects on monocyte/macrophage influx in the implants. Macrophages and GR1 monocytes were reduced in the treated group, whereas GR1 Low/Neg monocytes increased, as compared with the control group. Our findings are in agreement with some studies that showed that sodium propionate inhibits the pro-inflammatory actions of monocytes through the binding with the GPR41 receptor (mammalian G protein-coupled receptors), which has greater affinity for this short-chain fatty acid (SCFA) [49, 53, 54]. Fillipone et al. [24] demonstrated inhibition of murine macrophage activities by sodium propionate in *vitro and* attenuation of inflammation *in vivo* in a model of carrageenan-induced rat paw inflammation. The monocytes GR1^Low/Neg^, known as patrolling monocytes, are those that are related to the production of chemokines and the recruitment of cells to the site of inflammation [55–59]. We have previously reported the predominance of GR1 Low/Neg monocytes in our model [60]. Here, we confirm this finding and suggest a role of SP in modulating the differentiation of inflammatory macrophages in our model of FBR.

Mast cells are another cell type considered to be one of the main cells in fibrogenesis, due to their relevance in promoting inflammatory cell chemotaxis and in stimulating FBR events [1, 61–63]. Our results showed that SP reduced the number of this cell population as compared with the number in the control group. Studies show that inhibition of mastocytes and their products influence the intensity of inflammatory response and tissue remodeling. In a murine model deficient in mast cells, a significant reduction in the presence of phagocytes and fibrotic tissue was observed at the site of subcutaneous implants two weeks after biomaterial implantation. It was suggested that mast cell deficiency interfered with the recruitment of macrophages and neutrophils and their effects on FBR [3]. Other studies indicate that the permanence of mast cells at the implant site may be related to the degree of fibrotic encapsulation due to the secretion of TGF-β1 and proteases that stimulate fibroblasts and the formation of collagen fibers [61, 64]. Our results are in line with the notion that attenuated mastocytic infiltration interferes with the foreign body response.

Another important component of the tissue/implant interaction process is vascularization. It has been reported that angiogenesis establishes a relationship of co-dependence with inflammatory and fibrogenic events, promoting oxygen support and metabolites for the cells and tissues involved in both processes. At the sametime, it receives stimulation from the products of these events, so that inhibition of inflammation and its mediators can also suppress angiogenesis [65–67]. Among the molecules that stimulate angiogenesis, vascular endothelial growth factor (VEGF) is one of the main regulators of the process [68]. Published studies have shown that the action of SCFAs on VEGF production and angiogenesis varies according to the dose administered and the experimental model [69–71]. In a murine model of bronchopulmonary dysplasia, intraperitoneal administration of 1.2 mg/g of sodium propionate promoted angiogenesis [72]. In our FBR model, SP was able to reduce the number of blood vessels and VEGF levels in the fibrovascular tissue induced by the implants. To our knowledge, this is the first demonstration of the efficacy of a SCFA derivative in reducing these parameters in FBR.

The combination of FBGC and collagenous capsule, hallmarks of FBR, provides a barrier isolating the implants from the surrounding tissues which is detrimental to the implants’ function, safety, and biocompatibility [2, 8–11, 16]. In our study, we observed that the number of FBGCs was lower in the SP-treated group. Similarly, the treatment reduced the mean measurements of fibrovascular capsule thickness and total collagen production (type I and type III collagen) compared with control implants. Propionate has been shown to reduce cardiac fibrosis/remodeling in hypertensive mice [28], which is in line with our results. However, we found no study on the effects of SP on FBGCs. It was interesting to observe that type III collagen fibers were predominant in implants of the treated group, whereas type I was the main collagen found in the control group. It has been reported that decrease in remodeling intensity is favorable for better adaptation of the implant to the host [10, 73]. Thus, SP not only modulated the intensity of the fibrotic response induced by the implants, but also the nature of the response.

TGF-β pathway activation enhances fibrogenesis and remodeling in normal healing after tissue injury. It is also an important mediator of multiple fibrotic diseases and syndromes [74]. We reasoned that SP would be acting through the TGF-β pathway. Using western blot analysis and ELISA, we found a decrease in TGF-β1 phosphorylation and also in α-SMA protein and in the levels of TGF-β1 in the implants of the treated group. Thus, at least in part, SP exerted its effects through this pathway, which does not exclude the involvement of other pathways in the process. Alpha-SMA is an actin isoform associated with activation of fibroblast to myofibroblast. The phenotype of myofibroblast in expressing α-SMA and producing extracellular matrix compounds is regulated by transforming growth factor-beta (TGF-β) [75]. It is possible that SP may have acted on other remodeling markers present in the extracellular matrix of the newly formed fibrovascular tissue. For instance, metalloproteinases (MMP) and tissue inhibitors of MMPS (TIMPs) are known to regulate matrix turnover promoting the release of TGF-β sequestered within the ECM [76, 77]. Whether SP exerted its anti-fibrotic effects through modulation of MMPs on our *in vivo* model remains to be investigated.

Altogether, our results confirm and extend previous findings of the anti-inflammatory, anti-angiogenic and anti-fibrogenic actions of SP in different experimental models. However, this is the first evidence that this SCFA derivative is effective in attenuating FBR components, namely inflammatory cell recruitment/activation, blood vessels formation and key markers of fibrotic response (capsule thickness and FBGCs formation). Our data suggest that SP downregulated multiple axes involved in the process of adverse remodeling upon the interaction of foreign body material/host tissue. Thus, extending its range of actions to this adverse remodeling condition. Sodium propionate holds the potential for use as a therapeutic innovation in the treatment and/or prevention of complications inherent to the implantation of medical devices.

## Conclusions

Oral administration of sodium propionate modulated negatively the inflammatory phase, angiogenesis and foreign body reaction with consequent decrease in pathological remodeling in a murine model of polyether-polyurethane sponge implant.

Sodium propionate has the potential for use as a therapeutic innovation in the treatment of complications inherent to the implantation of implantable medical devices. However, more studies are needed to better understand the mechanisms involved in these processes.

## Supporting information

S1 File Manuscript data. Manuscript data are present in a table file. Table 1: Intra-implant leukocytes quantification. Table 2: Intra-implant inflammatory MPO enzyme and TNF-α cytokine quantification. Table 3: Intra-implant protein levels expression. Supplement Table 4: Histological quantification of fibrovascular tissue components per animal/slide.
(DOCX)

S1 Raw images Image of western blot gel. (PDF)

## References

[1] Zdolsek J, Eaton JW, Tang L. Histamine release and fibrinogen adsorption mediate acute inflammatory responses to biomaterial implants in humans. J Transl Med. 2007;5:31. doi: 10.1186/1479-5876-5-31 17603911 PMC1929055

[2] Anderson JM, Rodriguez A, Chang DT. Foreign body reaction to biomaterials. Semin Immunol. 2008;20(2):86–100. doi: 10.1016/j.smim.2007.11.004 18162407 PMC2327202

[3] Thevenot PT, Baker DW, Weng H, Sun MW, Tang L. The pivotal role of fibrocytes and mast cells in mediating fibrotic reactions to biomaterials. Biomaterials. 2011;32(33):8394–403. doi: 10.1016/j.biomaterials.2011.07.084 21864899 PMC3176925

[4] Pires ALR, & Moraes AMR. Biomaterials: types, applications, and market. Quim Nova,. 2015; v.38(n.7):957–71.

[5] Chu C, Liu L, Rung S, Wang Y, Ma Y, Hu C, et al. Modulation of foreign body reaction and macrophage phenotypes concerning microenvironment. J Biomed Mater Res A. 2020;108(1):127–35. doi: 10.1002/jbm.a.36798 31515867

[6] Doloff JC, Veiseh O, Vegas AJ, Tam HH, Farah S, Ma M, et al. Colony stimulating factor-1 receptor is a central component of the foreign body response to biomaterial implants in rodents and non-human primates. Nat Mater. 2017;16(6):671–80. 28319612 10.1038/nmat4866 PMC5445003

[7] Scheuermann K, Viana CTR, Dos Reis DC, de Lazari MGT, Orellano LAA, Machado CT, et al. Amitriptyline efficacy in decreasing implant-induced foreign body reaction. IUBMB Life. 2023;75(9):732–42. doi: 10.1002/iub.2725 37086464

[8] Gretzer C, Emanuelsson L, Liljensten E, Thomsen P. The inflammatory cell influx and cytokines changes during transition from acute inflammation to fibrous repair around implanted materials. J Biomater Sci Polym Ed. 2006;17(6):669–87. doi: 10.1163/156856206777346340 16892728

[9] Morais JM, Papadimitrakopoulos F, Burgess DJ. Biomaterials/tissue interactions: possible solutions to overcome foreign body response. AAPS J. 2010;12(2):188–96. doi: 10.1208/s12248-010-9175-3 20143194 PMC2844517

[10] Veiseh O, Vegas AJ. Domesticating the foreign body response: Recent advances and applications. Adv Drug Deliv Rev. 2019;144:148–61. doi: 10.1016/j.addr.2019.08.010 31491445 PMC6774350

[11] Carnicer-Lombarte A, Chen ST, Malliaras GG, Barone DG. Foreign Body Reaction to Implanted Biomaterials and Its Impact in Nerve Neuroprosthetics. Front Bioeng Biotechnol. 2021;9:622524. doi: 10.3389/fbioe.2021.622524 33937212 PMC8081831

[12] Patil SD, Papadmitrakopoulos F, Burgess DJ. Concurrent delivery of dexamethasone and VEGF for localized inflammation control and angiogenesis. J Control Release. 2007;117(1):68–79. doi: 10.1016/j.jconrel.2006.10.013 17169457

[13] Swartzlander MD, Barnes CA, Blakney AK, Kaar JL, Kyriakides TR, Bryant SJ. Linking the foreign body response and protein adsorption to PEG-based hydrogels using proteomics. Biomaterials. 2015;41:26–36. doi: 10.1016/j.biomaterials.2014.11.026 25522962 PMC4629245

[14] Chung L, Maestas DR, Jr., Housseau F, Elisseeff JH. Key players in the immune response to biomaterial scaffolds for regenerative medicine. Adv Drug Deliv Rev. 2017;114:184–92. doi: 10.1016/j.addr.2017.07.006 28712923

[15] Broly M, Marie J, Picard C, Demoures A, Raimbault C, Beylot-Barry M, et al. Management of granulomatous foreign body reaction to fillers with methotrexate. J Eur Acad Dermatol Venereol. 2020;34(4):817–20. doi: 10.1111/jdv.16027 31650637

[16] McNally AK, Anderson JM. Foreign body-type multinucleated giant cells induced by interleukin-4 express select lymphocyte co-stimulatory molecules and are phenotypically distinct from osteoclasts and dendritic cells. Exp Mol Pathol. 2011;91(3):673–81. doi: 10.1016/j.yexmp.2011.06.012 21798256 PMC3220734

[17] Vinolo MA, Ferguson GJ, Kulkarni S, Damoulakis G, Anderson K, Bohlooly YM, et al. SCFAs induce mouse neutrophil chemotaxis through the GPR43 receptor. PLoS One. 2011;6(6): e21205. doi: 10.1371/journal.pone.0021205 21698257 PMC3115979

[18] Koh A, De Vadder F, Kovatcheva-Datchary P, Backhed F. From Dietary Fiber to Host Physiology: Short-Chain Fatty Acids as Key Bacterial Metabolites. Cell. 2016;165(6):1332–45. doi: 10.1016/j.cell.2016.05.041 27259147

[19] Silva LG, Ferguson BS, Avila AS, Faciola AP. Sodium propionate and sodium butyrate effects on histone deacetylase (HDAC) activity, histone acetylation, and inflammatory gene expression in bovine mammary epithelial cells. J Anim Sci. 2018;96(12):5244–52. doi: 10.1093/jas/sky373 30252114 PMC6276571

[20] Ratajczak W, Ryl A, Mizerski A, Walczakiewicz K, Sipak O, Laszczynska M. Immunomodulatory potential of gut microbiome-derived short-chain fatty acids (SCFAs). Acta Biochim Pol. 2019;66(1):1–12. 30831575 10.18388/abp.2018_2648

[21] Castro PR, Bittencourt LFF, Larochelle S, Andrade SP, Mackay CR, Slevin M, et al. GPR43 regulates sodium butyrate-induced angiogenesis and matrix remodeling. Am J Physiol Heart Circ Physiol. 2021;320(3): H1066–H79. doi: 10.1152/ajpheart.00515.2019 33356962

[22] De Lazari MGT, Pereira LX, Orellano LAA, Scheuermann K, Machado CT, Vasconcelos AC, et al. Sodium Butyrate Downregulates Implant-Induced Inflammation in Mice. Inflammation. 2020;43(4):1259–68. doi: 10.1007/s10753-020-01205-0 32125592

[23] De Lazari MGT, Viana CTR, Pereira LX, Orellano LAA, Ulrich H, Andrade SP, et al. Sodium butyrate attenuates peritoneal fibroproliferative process in mice. Exp Physiol. 2023;108(1):146–57. doi: 10.1113/EP090559 36459573 PMC10103766

[24] Filippone A, Lanza M, Campolo M, Casili G, Paterniti I, Cuzzocrea S, et al. The Anti-Inflammatory and Antioxidant Effects of Sodium Propionate. Int J Mol Sci. 2020;21(8). doi: 10.3390/ijms21083026 32344758 PMC7215993

[25] Tong LC, Wang Y, Wang ZB, Liu WY, Sun S, Li L, et al. Propionate Ameliorates Dextran Sodium Sulfate-Induced Colitis by Improving Intestinal Barrier Function and Reducing Inflammation and Oxidative Stress. Front Pharmacol. 2016;7:253. doi: 10.3389/fphar.2016.00253 27574508 PMC4983549

[26] Wang J, Wei Z, Zhang X, Wang Y, Yang Z, Fu Y. Propionate Protects against Lipopolysaccharide-Induced Mastitis in Mice by Restoring Blood-Milk Barrier Disruption and Suppressing Inflammatory Response. Front Immunol. 2017;8:1108. doi: 10.3389/fimmu.2017.01108 28966615 PMC5605562

[27] Chen D, Gao ZQ, Wang YY, Wan BB, Liu G, Chen JL, et al. Sodium Propionate Enhances Nrf2-Mediated Protective Defense Against Oxidative Stress and Inflammation in Lipopolysaccharide-Induced Neonatal Mice. J Inflamm Res. 2021;14:803–16. doi: 10.2147/JIR.S303105 33732006 PMC7957230

[28] Bartolomaeus H, Balogh A, Yakoub M, Homann S, Marko L, Hoges S, et al. Short-Chain Fatty Acid Propionate Protects From Hypertensive Cardiovascular Damage. Circulation. 2019;139(11):1407–21. doi: 10.1161/CIRCULATIONAHA.118.036652 30586752 PMC6416008

[29] Pereira LX, Viana CTR, Orellano LAA, de Almeida SA, de Lazari MGT, Couto LC, et al. Kinetics of pancreatic tissue proliferation in a polymeric platform in mice. Pancreatology. 2018;18(2):221–9. doi: 10.1016/j.pan.2017.12.011 29289464

[30] Jesus ICG, Araujo FM, Mesquita T, Junior NNS, Silva MM, Morgan HJN, et al. Molecular basis of Period 1 regulation by adrenergic signaling in the heart. FASEB J. 2021;35(10): e21886. doi: 10.1096/fj.202100441R 34473369

[31] Oviedo-Socarras T, Vasconcelos AC, Barbosa IX, Pereira NB, Campos PP, Andrade SP. Diabetes alters inflammation, angiogenesis, and fibrogenesis in intraperitoneal implants in rats. Microvasc Res. 2014;93:23–9. doi: 10.1016/j.mvr.2014.02.011 24594441

[32] Vollkommer T, Henningsen A, Friedrich RE, Felthaus OH, Eder F, Morsczeck C, et al. Extent of Inflammation and Foreign Body Reaction to Porous Polyethylene In Vitro and In Vivo. In Vivo. 2019;33(2):337–47. doi: 10.21873/invivo.11479 30804110 PMC6506308

[33] Li M, van Esch B, Henricks PAJ, Garssen J, Folkerts G. Time and Concentration Dependent Effects of Short Chain Fatty Acids on Lipopolysaccharide- or Tumor Necrosis Factor alpha-Induced Endothelial Activation. Front Pharmacol. 2018;9:233. 29615908 10.3389/fphar.2018.00233 PMC5867315

[34] Vestweber D. How leukocytes cross the vascular endothelium. Nat Rev Immunol. 2015;15(11):692–704. doi: 10.1038/nri3908 26471775

[35] Hu C, Zeng D, Huang Y, Deng Q, Liu S, Zhou W, et al. Sodium Butyrate Ameliorates Atopic Dermatitis-Induced Inflammation by Inhibiting HDAC3-Mediated STAT1 and NF-kappaB Pathway. Inflammation. 2024;47(3):989–1001. 38159175 10.1007/s10753-023-01955-7

[36] Castro PR, Marques SM, Viana CT, Campos PP, Ferreira MA, Barcelos LS, et al. Deletion of the chemokine receptor CCR2 attenuates foreign body reaction to implants in mice. Microvasc Res. 2014;95:37–45. doi: 10.1016/j.mvr.2014.07.002 25020267

[37] Kyriakides TR, Kim HJ, Zheng C, Harkins L, Tao W, Deschenes E. Foreign body response to synthetic polymer biomaterials and the role of adaptive immunity. *Biomed Mater*. 2022;17(2):022007. doi: 10.1088/1748-605X/ac5574 35168213 PMC9159526

[38] Avula MN, Rao AN, McGill LD, Grainger DW, Solzbacher F. Foreign body response to subcutaneous biomaterial implants in a mast cell-deficient Kit(w-Sh) murine model. Acta Biomater. 2014;10(5):1856–63. doi: 10.1016/j.actbio.2013.12.056 24406200

[39] Soehnlein O, Steffens S, Hidalgo A, Weber C. Neutrophils as protagonists and targets in chronic inflammation. Nat Rev Immunol. 2017;17(4):248–61. doi: 10.1038/nri.2017.10 28287106

[40] Selders GS, Fetz AE, Radic MZ, Bowlin GL. An overview of the role of neutrophils in innate immunity, inflammation and host-biomaterial integration. Regen Biomater. 2017;4(1):55–68. doi: 10.1093/rb/rbw041 28149530 PMC5274707

[41] Wang H, Pan L, Liu Z. Neutrophils as a Protagonist and Target in Chronic Rhinosinusitis. Clin Exp Otorhinolaryngol. 2019;12(4):337–47. doi: 10.21053/ceo.2019.00654 31394895 PMC6787473

[42] Maslowski KM, Vieira AT, Ng A, Kranich J, Sierro F, Yu D, et al. Regulation of inflammatory responses by gut microbiota and chemoattractant receptor GPR43. Nature. 2009;461(7268):1282–6. doi: 10.1038/nature08530 19865172 PMC3256734

[43] Vinolo MA, Rodrigues HG, Nachbar RT, Curi R. Regulation of inflammation by short chain fatty acids. Nutrients. 2011;3(10):858–76. doi: 10.3390/nu3100858 22254083 PMC3257741

[44] Correa RO, Vieira A, Sernaglia EM, Lancellotti M, Vieira AT, Avila-Campos MJ, et al. Bacterial short-chain fatty acid metabolites modulate the inflammatory response against infectious bacteria. Cell Microbiol. 2017;19(7). doi: 10.1111/cmi.12720 28070968

[45] Jhunjhunwala S, Aresta-DaSilva S, Tang K, Alvarez D, Webber MJ, Tang BC, et al. Neutrophil Responses to Sterile Implant Materials. PLoS One. 2015;10(9): e0137550. doi: 10.1371/journal.pone.0137550 26355958 PMC4565661

[46] Mariani E, Lisignoli G, Borzi RM, Pulsatelli L. Biomaterials: Foreign Bodies or Tuners for the Immune Response? Int J Mol Sci. 2019;20(3). doi: 10.3390/ijms20030636 30717232 PMC6386828

[47] Ode Boni BO, Lamboni L, Souho T, Gauthier M, Yang G. Immunomodulation and cellular response to biomaterials: the overriding role of neutrophils in healing. Materials Horizons. 2019;6(6):1122–37.

[48] Brodbeck WG, Macewan M, Colton E, Meyerson H, Anderson JM. Lymphocytes and the foreign body response: lymphocyte enhancement of macrophage adhesion and fusion. J Biomed Mater Res A. 2005;74(2):222–9. doi: 10.1002/jbm.a.30313 15948198

[49] Chang DT, Colton E, Anderson JM. Paracrine and juxtacrine lymphocyte enhancement of adherent macrophage and foreign body giant cell activation. J Biomed Mater Res A. 2009;89(2):490–8. doi: 10.1002/jbm.a.31981 18437695 PMC3864690

[50] Brown AJ, Goldsworthy SM, Barnes AA, Eilert MM, Tcheang L, Daniels D, et al. The Orphan G protein-coupled receptors GPR41 and GPR43 are activated by propionate and other short chain carboxylic acids. J Biol Chem. 2003;278(13):11312–9. doi: 10.1074/jbc.M211609200 12496283

[51] Park J, Kim M, Kang SG, Jannasch AH, Cooper B, Patterson J, et al. Short-chain fatty acids induce both effector and regulatory T cells by suppression of histone deacetylases and regulation of the mTOR-S6K pathway. Mucosal Immunol. 2015;8(1):80–93. doi: 10.1038/mi.2014.44 24917457 PMC4263689

[52] Garg K, Pullen NA, Oskeritzian CA, Ryan JJ, Bowlin GL. Macrophage functional polarization (M1/M2) in response to varying fiber and pore dimensions of electrospun scaffolds. Biomaterials. 2013;34(18):4439–51. doi: 10.1016/j.biomaterials.2013.02.065 23515178 PMC3623371

[53] Cox MA, Jackson J, Stanton M, Rojas-Triana A, Bober L, Laverty M, et al. Short-chain fatty acids act as antiinflammatory mediators by regulating prostaglandin E(2) and cytokines. World J Gastroenterol. 2009;15(44):5549–57. doi: 10.3748/wjg.15.5549 19938193 PMC2785057

[54] Parada Venegas D, De la Fuente MK, Landskron G, Gonzalez MJ, Quera R, Dijkstra G, et al. Short Chain Fatty Acids (SCFAs)-Mediated Gut Epithelial and Immune Regulation and Its Relevance for Inflammatory Bowel Diseases. Front Immunol. 2019;10:277. doi: 10.3389/fimmu.2019.00277 30915065 PMC6421268

[55] Serbina NV, Jia T, Hohl TM, Pamer EG. Monocyte-mediated defense against microbial pathogens. Annu Rev Immunol. 2008;26:421–52. doi: 10.1146/annurev.immunol.26.021607.090326 18303997 PMC2921669

[56] Auffray C, Sieweke MH, Geissmann F. Blood monocytes: development, heterogeneity, and relationship with dendritic cells. Annu Rev Immunol. 2009;27:669–92. doi: 10.1146/annurev.immunol.021908.132557 19132917

[57] Ziegler-Heitbrock L, Ancuta P, Crowe S, Dalod M, Grau V, Hart DN, et al. Nomenclature of monocytes and dendritic cells in blood. Blood. 2010;116(16): e74–80. doi: 10.1182/blood-2010-02-258558 20628149

[58] Ingersoll MA, Spanbroek R, Lottaz C, Gautier EL, Frankenberger M, Hoffmann R, et al. Comparison of gene expression profiles between human and mouse monocyte subsets. Blood. 2010;115(3): e10–9. doi: 10.1182/blood-2009-07-235028 19965649 PMC2810986

[59] Crane MJ, Daley JM, van Houtte O, Brancato SK, Henry WL, Jr., Albina JE. The monocyte to macrophage transition in the murine sterile wound. PLoS One. 2014;9(1): e86660. doi: 10.1371/journal.pone.0086660 24466192 PMC3899284

[60] Rodrigues Viana CT, Castro PR, Marques SM, Paz Lopes MT, Goncalves R, Campos PP, et al. Differential Contribution of Acute and Chronic Inflammation to the Development of Murine Mammary 4T1 Tumors. PLoS One. 2015;10(7): e0130809. doi: 10.1371/journal.pone.0130809 26158775 PMC4497676

[61] Tcacencu I, Wendel M. Collagen-hydroxyapatite composite enhances regeneration of calvaria bone defects in young rats but postpones the regeneration of calvaria bone in aged rats. J Mater Sci Mater Med. 2008;19(5):2015–21. doi: 10.1007/s10856-007-3284-2 17952564

[62] Weiskirchen R, Meurer SK, Liedtke C, Huber M. Mast Cells in Liver Fibrogenesis. Cells. 2019;8(11). doi: 10.3390/cells8111429 31766207 PMC6912398

[63] Ozpinar EW, Frey AL, Cruse G, Freytes DO. Mast Cell-Biomaterial Interactions and Tissue Repair. Tissue Eng Part B Rev. 2021;27(6):590–603. doi: 10.1089/ten.TEB.2020.0275 33164714 PMC8739845

[64] Larouche J, Sheoran S, Maruyama K, Martino MM. Immune Regulation of Skin Wound Healing: Mechanisms and Novel Therapeutic Targets. Adv Wound Care (New Rochelle). 2018;7(7):209–31. doi: 10.1089/wound.2017.0761 29984112 PMC6032665

[65] Carmeliet P. Manipulating angiogenesis in medicine. J Intern Med. 2004;255(5):538–61. doi: 10.1111/j.1365-2796.2003.01297.x 15078497

[66] Crewe C, An YA, Scherer PE. The ominous triad of adipose tissue dysfunction: inflammation, fibrosis, and impaired angiogenesis. J Clin Invest. 2017;127(1):74–82. doi: 10.1172/JCI88883 28045400 PMC5199684

[67] Kastellorizios M, Papadimitrakopoulos F, Burgess DJ. Prevention of foreign body reaction in a pre-clinical large animal model. J Control Release. 2015;202:101–7. doi: 10.1016/j.jconrel.2015.01.038 25645376

[68] Keck PJ, Hauser SD, Krivi G, Sanzo K, Warren T, Feder J, et al. Vascular permeability factor, an endothelial cell mitogen related to PDGF. Science. 1989;246(4935):1309–12. doi: 10.1126/science.2479987 2479987

[69] Sawa H, Murakami H, Ohshima Y, Murakami M, Yamazaki I, Tamura Y, et al. Histone deacetylase inhibitors such as sodium butyrate and trichostatin A inhibit vascular endothelial growth factor (VEGF) secretion from human glioblastoma cells. Brain Tumor Pathol. 2002;19(2):77–81. doi: 10.1007/BF02478931 12622137

[70] Belakavadi M, Prabhakar BT, Salimath BP. Butyrate-induced proapoptotic and antiangiogenic pathways in EAT cells require activation of CAD and downregulation of VEGF. Biochem Biophys Res Commun. 2005;335(4):993–1001. doi: 10.1016/j.bbrc.2005.07.172 16105646

[71] Liu D, Andrade SP, Castro PR, Treacy J, Ashworth J, Slevin M. Low Concentration of Sodium Butyrate from Ultrabraid+NaBu suture, Promotes Angiogenesis and Tissue Remodelling in Tendon-bones Injury. Sci Rep. 2016;6:34649. doi: 10.1038/srep34649 27694930 PMC5046145

[72] Chen G, Ran X, Li B, Li Y, He D, Huang B, et al. Sodium Butyrate Inhibits Inflammation and Maintains Epithelium Barrier Integrity in a TNBS-induced Inflammatory Bowel Disease Mice Model. EBioMedicine. 2018;30:317–25. doi: 10.1016/j.ebiom.2018.03.030 29627390 PMC5952406

[73] Chandorkar Y, Bhaskar N, Madras G, Basu B. Long-term sustained release of salicylic acid from cross-linked biodegradable polyester induces a reduced foreign body response in mice. Biomacromolecules. 2015;16(2):636–49. doi: 10.1021/bm5017282 25559641

[74] Branton MH, Kopp JB. TGF-beta and fibrosis. Microbes Infect. 1999;1(15):1349–65. doi: 10.1016/s1286-4579(99)00250-6 10611762

[75] Grotendorst GR, Rahmanie H, Duncan MR. Combinatorial signaling pathways determine fibroblast proliferation and myofibroblast differentiation. FASEB J. 2004;18(3):469–79. doi: 10.1096/fj.03-0699com 15003992

[76] Yo Q, Stamenkovic I. Cell surface-localized matrix metalloproteinase-9 proteolytically activates TGF-beta and promotes tumor invasion and angiogenesis. *Genes Dev*. 2000;14:163–176. 10652271 PMC316345

[77] Page-McCaw A, Ewald AJ, Werb Z. Matrix metalloproteinases and the regulation of tissue remodeling. *Nat Rev Mol Cell Biol*. 2007;8:221–233. 17318226 10.1038/nrm2125 PMC2760082

