# Supplementary material for: Sodium propionate decreases implant-induced foreign body response in mice
Source: PLoS One. 2025 Feb 19;20(2):e0316764. doi: 10.1371/journal.pone.0316764 (PMC11838875; doi:10.1371/journal.pone.0316764)
Supplement: S1 File — Manuscript data are present in a table file. Table 1: Intra-implant leukocytes quantification. Table 2: Intra-implant inflammatory MPO enzyme and TNF-α cytokine quantification. Table 3: Intra-implant protein levels expression. Supplement Table 4: Histological quantification of fibrovascular tissue components per animal/slide. (DOCX) [file pone.0316764.s001.docx]

**DATA**

Table 1: Intra-implant leukocytes quantification

|  | **Flow Cytometry** | | | | |
| --- | --- | --- | --- | --- | --- |
|  |  |  |  |  |  |
|  | CTL Lymphocytes (CD45 + CD3) | SP -Lymphocytes (CD45 + CD3) |  | Control neutrophils (GR1 + F4 / 80^Neg^) | SP neutrophis  (GR1+F4/80^Neg^) |
| 1 | 7.81 | 5.84 |  | 1.81 | 0.16 |
| 2 | 10.6 | 6.74 |  | 1.55 | 0.3 |
| 3 | 10.8 | 6.94 |  | 1.4 | 1.85 |
| 4 | 8.67 | 8.64 |  | 1.96 | 1.2 |
| 5 | 10.9 | 10.5 |  | 0.85 | 0.31 |
| 6 | 8.46 | 6.62 |  | 1.11 | 0.11 |
| 7 | 9.7 | 8 |  | 2.36 | 0.15 |
| 8 | 9.12 | 6.71 |  | 1.52 | 0.47 |
| 9 | outlier | 9.34 |  | 4.26 | 0.25 |

|  | Control macrocrophages (F4/ 80 ^High^ GR1^Low/Neg^) | Propionate macrocrophages (F4/ 80 ^High^ GR1^Low/Neg^) |  | Control monocytes (F4 / 80 + CD11b + GR1^Low^) | Propionate monocytes (F4 / 80 + CD11b + GR1^Low^) |  | Control monocytes (F4 / 80 + CD11b + GR1^High^) | Propionate monocytes (F4 / 80 + CD11b + GR1^High^) |
| --- | --- | --- | --- | --- | --- | --- | --- | --- |
| 1 | 40.2 | 31.3 |  | 16.2 | 40.8 |  | 33.6 | 43.4 |
| 2 | 44.2 | 52.7 |  | 36.1 | 31.3 |  | 22.3 | 32.1 |
| 3 | 42.2 | 54 |  | 37.9 | 33.3 |  | 49.3 | 45.1 |
| 4 | 40.6 | 54.7 |  | 32.8 | 23.9 |  | 51.5 | 35.3 |
| 5 | 44.8 | 4.59 |  | 38.7 | 47.3 |  | 35 | 7.54 |
| 6 | 40.2 | 4.18 |  | 38.3 | 59.8 |  | 43.2 | 23 |
| 7 | 40.1 | 1.44 |  | 32.1 | 51.8 |  | 55.2 | 15.2 |
| 8 | 42.2 | 2.71 |  | 36.8 | 54.3 |  | 35.9 | 7.71 |
| 9 | 39.3 | 1.8 |  | 31.1 | 52.6 |  | 43.1 | 17 |
| 10 | 37.8 | 4.42 |  | outlier | 60 |  | 18.5 | 9.79 |
| Supplement Table 2: Intra-implant inflammatory MPO enzime and TNF-α cytokine quantification   \| **MPO dosage** \| \| \| \| --- \| --- \| --- \| \|  \| CTL \| SP \| \| 1 \| 5.6 \| 2.75 \| \| 2 \| 3.76 \| 2.27 \| \| 3 \| 3.64 \| 2.5 \| \| 4 \| 2.57 \| 2.01 \| \| 5 \| 2.09 \| 3.22 \| \| 6 \| 2.23 \| 2.26 \| \| 7 \| 2.43 \| 2.23 \| \| 8 \| 2.47 \| 2.26 \| \| 9 \| 3.7 \| 2.23 \| \|  \| **TNF-α levels** \| \| \|  \| CTL \| SP \| \| 1 \| 0.181997 \| 0.066551 \| \| 2 \| 0.21783 \| 0.020657 \| \| 3 \| 0.034287 \| 0.030724 \| \| 4 \| 0.026178 \| 0.024103 \| \| 5 \| 0.031334 \| 0.019725 \| \| 6 \| 0.171082 \| 0.016279 \| \| 7 \| 0.11141 \| 0.007971 \| \| 8 \| 0.05326 \| 0.038043 \| \| 9 \| outlier \| outlier \| \|  \| **VEGF levels** \| \| \|  \| CTL \| SP \| \| 1 \| 0.1029971 \| 0.07149477 \| \| 2 \| 1.016057 \| 0.326542 \| \| 3 \| 1.529718 \| 0.049645 \| \| 4 \| 0.2097431 \| 0.00394 \| \| 5 \| 0.7095238 \| 0 \| \|  \| **TGF-β 1 levels** \| \| \|  \| CTL \| SP \| \| 1 \| 2.930612 \| 0.4031153 \| \| 2 \| 1.216681 \| 0.8104893 \| \| 3 \| 1.5756 \| 0.5833787 \| \| 4 \| 2.287994 \| 0.3022225 \| \| 5 \| 0.55379 \| 0.4481737 \| \| 6 \| 0.824518 \| 0.2080278 \| \| 7 \| 0.7947522 \| 0.457356 \| \| 8 \| 0.5432308 \| 0.027332 \| | | | | | | | | |

Table 3: Intra- Protein levels expression.

| **α-SMA** | | |
| --- | --- | --- |
|  | CTL | SP |
| 1 | 1.570245 | 0.6802676 |
| 2 | 0.6820055 | 0.5412527 |
| 3 | 0.9409851 | 0.5379328 |
| 4 | 0.806764 | 0.5420476 |
| 5 | 1.059706 | 0.7048131 |
| 6 | 1.059706 | 0.997266 |
| 7 | 1.506147 | 0.7268619 |
| 8 | 0.6144949 | 0.7928337 |
|  | **TGF-β 1** | |
|  | CTL | SP |
| 1 | 0.6305943 | 0.9072827 |
| 2 | 1.688359 | 1.094958 |
| 3 | 1.463211 | 0.5859033 |
| 4 | 2.362253 | 1.20973 |
| 5 | 0.8147264 | 0.8582115 |
| 6 | 1.250912 | 0.1303185 |
| 7 | 1.33 | 0.08564721 |
| 8 | outlier | 1.060098 |

Table 4 : Histological quantification of fibrovascular tissue components per animal/slide.

| **Mumber of Mast cells per slide/animal 30 fields per slide** | | |
| --- | --- | --- |
|  | CTL | SP |
| 1 | 3.305555556 | 3.666666667 |
| 2 | 5.074074074 | 3.148148148 |
| 3 | 4.740740741 | 3.740740741 |
| 4 | 3.814814815 | 2.259259259 |
| 5 | 4.64 | 1.259259259 |
| **Number of vessels/slide 30 fields per slide** | | |
|  | CTL | SP |
| 1 | 8.825 | 4.325 |
| 2 | 4.816666 | 1.975 |
| 3 | 5.525 | 0 |
| 4 | 8.0406504 | 0 |
| 5 | 4.8139534 | 0 |
| **Number of Multinucleated giant cells/slide 30 fields per slide** | | |
|  | CTL | SP |
| 1 | 3.5 | 1.5 |
| 2 | 3.518 | 1.9 |
| 3 | 4.482 | 0.5364 |
| 4 | 3.555 | 1.5 |
| 5 | 4.723 | 0 |
| **Capsule thickness /slide 5 fields per slide/area μm2** | | |
|  | CTL | SP |
| 1 | 387.8717333 | 191.0323667 |
| 2 | 254.5491333 | 205.4098333 |
| 3 | 178.9451667 | 277.0260333 |
| 4 | 424.5503333 | 160.6317333 |
| 5 | 311.5 | 140 |
| **Total Collagen/ slide 50 fields per slide/ area μm2** | | |
|  | CTL | SP |
| 1 | 2183627.5 | 1292267.4 |
| 2 | 4564180.763 | 1156764.917 |
| 3 | 1700853.111 | 2051809.282 |
| 4 | 5242795.66 | 135850 |
| 5 | 1900952.122 | 135850 |
| **Type I Collagen** | | |
|  | CTL | SP |
| 1 | 1299821.061 | 239140.483 |
| 2 | 1990047.485 | 200063.671 |
| 3 | 1169144.833 | 15823.02542 |
| 4 | 1542702.321 | 804838.2381 |
| 5 | 819772.1864 | 291858.4634 |
|  | **Type III Collagen** | |
|  | CTL | SP |
| 1 | 855808.917 | 1460881.926 |
| 2 | 2337567.678 | 1434115.662 |
| 3 | 1315993.167 | 399610.246 |
| 4 | 863966.3483 | 294378.3333 |
| 5 | 545690.3421 | 1460881.926 |

Statistical analysis

All data were analyzed using GraphPad Prism for Windows (GraphPad Software Inc.). Results are expressed as mean ± SEM or Median. Comparisons between Two groups were made using Unpaired t test or Mann Whitney test. Differences between means were considered significant when p values were <0.05.
