# Supplementary material for: Sodium propionate decreases implant-induced foreign body response in mice
Source: PLoS One. 2025 Feb 19;20(2):e0316764. doi: 10.1371/journal.pone.0316764 (PMC11838875; doi:10.1371/journal.pone.0316764)

α-SMA (50 kDa)

Fig 6a

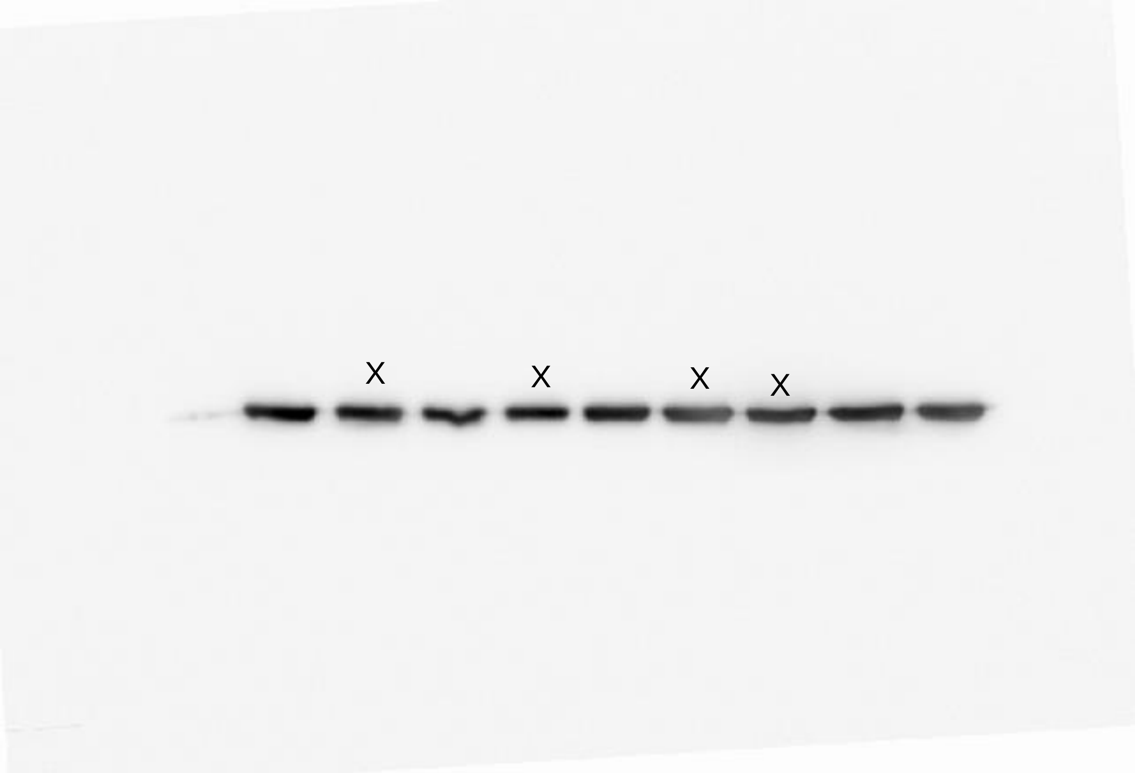

Control Propionate

R1 R2 R3 R4 R5 R6 R7 R8 R9

The 'X' above the lanes indicates those used in the final figure.

TGF-β1 (25kDa)

Fig 6a

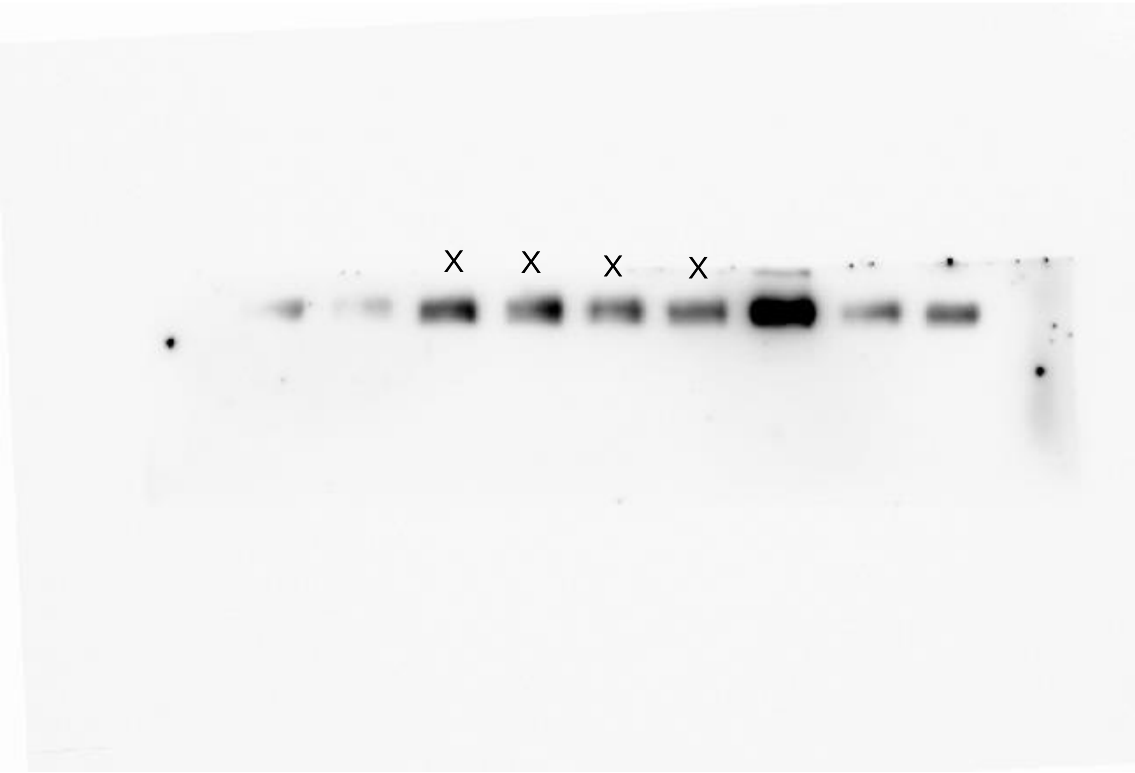

Control Propionate

R1 R2 R3 R4 R5 R6 R7 R8 R9

The 'X' above the lanes indicates those used in the final figure.

GAPDH (37kDa – Red Arrow)

Fig 6a

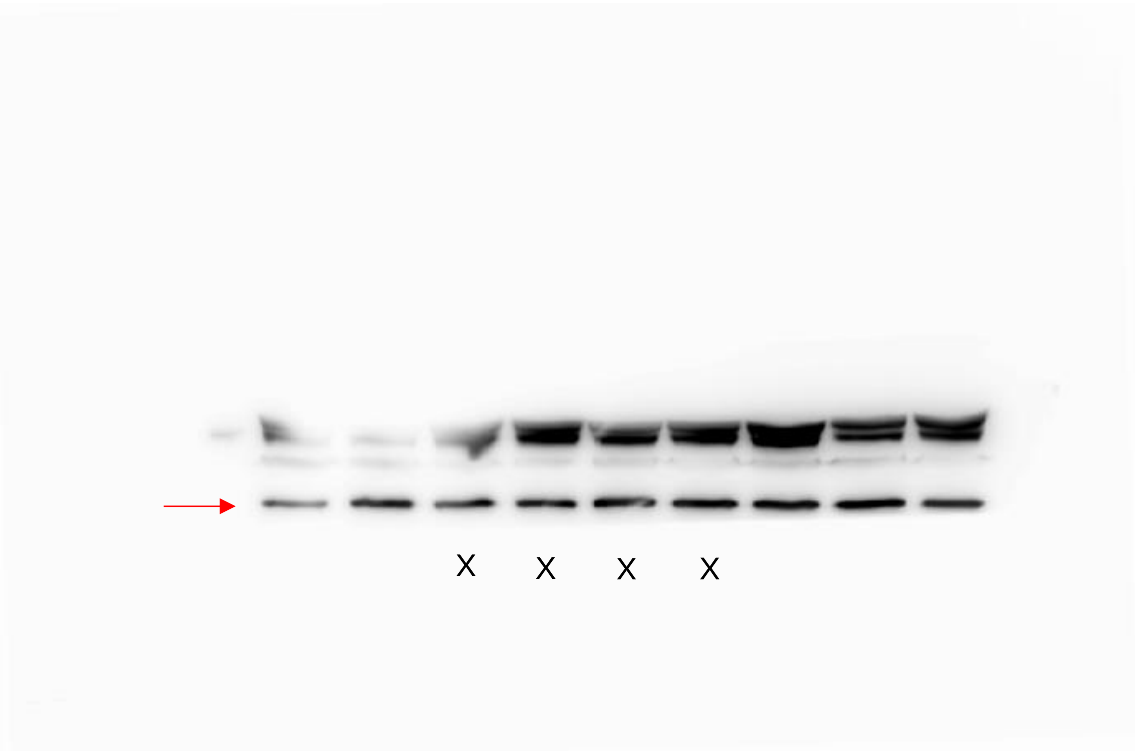

The 'X' below the lanes indicates those used in the final figure.

Generated Figure Panel

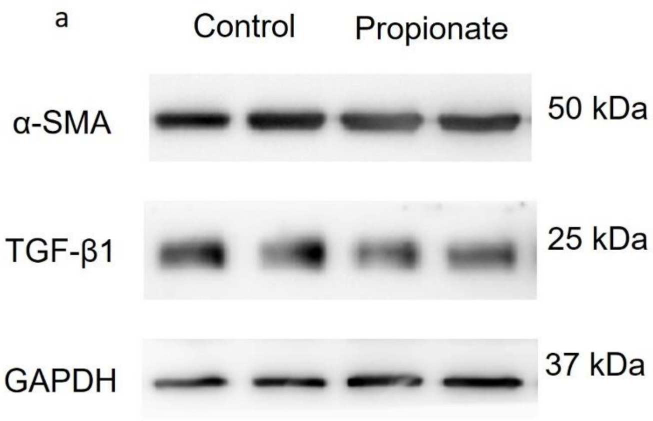

Supplement: S1 Raw images — (PDF) [file pone.0316764.s002.pdf]
